# Genome Assembly of Methicillin-Resistant Quality Control Strain *Staphylococcus aureus* CDC73-57501 (ATCC 29247)

**DOI:** 10.1128/genomeA.00961-14

**Published:** 2014-10-02

**Authors:** H. E. Daligault, K. W. Davenport, T. D. Minogue, K. A. Bishop-Lilly, S. M. Broomall, D. C. Bruce, P. S. Chain, S. R. Coyne, T. Freitas, K. G. Frey, H. S. Gibbons, J. Jaissle, C.-C. Lo, L. Meincke, A. C. Munk, C. L. Redden, C. N. Rosenzweig, S. L. Johnson

**Affiliations:** a Los Alamos National Laboratory (LANL), Los Alamos, New Mexico, USA; b Diagnostic Systems Division (DSD), United States Army Medical Research Institute of Infectious Diseases (USAMRIID), Fort Detrick, Maryland, USA; c Naval Medical Research Center (NMRC)-Frederick, Fort Detrick, Maryland, USA; d Henry M. Jackson Foundation, Bethesda, Maryland, USA; e United States Army Edgewood Chemical Biological Center (ECBC), Aberdeen Proving Ground, Aberdeen, Maryland, USA

## Abstract

*Staphylococcus aureus* is a major cause of bacterial infections in the United States, with high percentages of serious infections resistant to a variety of β-lactam antibiotics. Here, we present the scaffolded genome assembly into 16 contigs of *S. aureus* CDC73-57501 (ATCC 29247), a methicillin-resistant quality control strain.

## GENOME ANNOUNCEMENT

Both a commensal and a pathogen, *Staphyloccus aureus* is a well-known Gram-positive bacterium that often inhabits human skin and mucosa (1, 2). This continual contact with human skin is often blamed for the antibiotic resistance developed by so many staphylococci (3, 4). Strains resistant to all β-lactams, often referred to simply as community-acquired methicillin-resistant *S. aureus* (CA-MRSA), have become common among U.S. urban populations since the 1980s (5). A recent report by the U.S. Centers for Disease Control and Prevention listed MRSA as causing nearly 80,500 severe infections and 11,300 deaths in 2011 alone (6). Here, we present a scaffolded genome assembly of *S. aureus* CDC73-57501 (ATCC 29247), a methicillin-resistant strain used in diagnostic and quality control measures.

High-quality genomic DNA was extracted from purified isolates of each strain using a Qiagen Genomic-tip 500 at the USAMRIID Diagnostic Systems Division (DSD). Specifically, a 100-mL bacterial culture was grown to stationary phase and nucleic acid was extracted per the manufacturer’s recommendations. Sequence data generated included a combination of Illumina and 454 technologies (7, 8). For each genome, we constructed and sequenced an Illumina library of 100-bp reads at 669-fold genome coverage and a separate long-insert paired-end library (6,988 ± 1,747-bp insert and 14-fold genome coverage) (Roche 454 Titanium platform). The two libraries were assembled together in Newbler (version 2.6; Roche), and the consensus sequences were computationally shredded into 2-kbp overlapping fake reads (shreds). The raw reads were also assembled in Velvet (version 1.2.08), and those consensus sequences were computationally shredded into 1.5-kbp overlapping shreds (9). Draft data from all platforms were then assembled together with Allpaths (version 39750), and the consensus sequences were computationally shredded into 10-kbp overlapping shreds (10). We then integrated the Newbler consensus shreds, Velvet consensus shreds, Allpaths consensus shreds, and a subset of the long-insert read pairs using parallel Phrap (version SPS-4.24; High Performance Software, LLC). Possible misassemblies were corrected and some gap closure accomplished with manual editing in Consed (11–13).

Automatic annotation of the *S. aureus* 73-57501 genome utilized an Ergatis-based workflow at LANL with minor manual curation. The final assembly is 2,927,712 bp long (32.9% G+C content) with 2,778 coding, 7 rRNA, and 58 tRNA sequences. Preliminary review revealed *mecI* (methicillin resistance), *tetM* (tetracycline resistance), and three penicillin binding proteins, as well as the regulator *agrA* often associated with virulence (14).

### Nucleotide sequence accession number.

The annotated genome assembly of *S. aureus* 73-57501 is available in GenBank under the accession number JOVN00000000.
